# Chicago Medical Society

**Published:** 1867-06

**Authors:** 


### Proceedings of Societies.

### CHICAGO MEDICAL SOCIETY.

The regular annual meeting of the Society was held on the evening of April 7th. The following members were chosen officers for the ensuing year:—

*President*—J. P. Ross, M.D.

*Vice-President*—John Reid, M.D.

*Secretary and Treasurer*—H. M. Lyman, M.D.

*Censors*—E. L. Holmes, M.D., John Reid, M.D., Thomas Bevan, M.D.

*Committee on Ethics*—S. Wickersham, M.D., G. C. Paoli, M.D., T. D. Fitch, M.D.

*Committee on Sanitary Condition of City*—N. S. Davis, M.D., R. G. Bogue, M.D., R. M. Lackey, M.D.

At no time since its organization, has the Society been in a more encouraging condition than during the past year. The weekly meetings have been regularly held and well attended. The activity of the Society has been increased by the addition of a large number of new members. Since the opening of the

Cook County Hospital, the meetings have been specially instructive, not only on account of the interesting cases which have been reported, but particularly on account of the large number of pathological specimens which have been presented.

It has been stated by those who are acquainted with the condition of medical societies in other large cities, that this Society compares most favorably with similar organizations elsewhere, as regards number of members in attendance, and interest of discussions and reports.

### SYPHILITIC TESTICLE.

Dr. C. G. Smith exhibited a specimen of benign fungus in a syphilitic testis. The patient had primary syphilis seven years ago. Two years ago he was affected with rupiæ, induration, and enlargement of the testicle, on the anterior portion of which there soon appeared an ulcer an inch in diameter. This ulcer soon healed under the use of the ordinary antisyphilitic remedies. In a few weeks the ulceration reäppeared, but was neglected by the patient for nearly two years. There was never much pain or inconvenience, the patient being able to attend to his occupation during the whole time. The growth of the fungus was very slow, finally, however, involving the whole anterior half of the organ. Medication failed to arrest the disease.

### A CASE OF HYDROPHOBIA.

Prof. Nelson related the history of a case of hydrophobia, in a boy 15 years of age, who was bitten on the lip November 27, 1866. The wound healed readily. On the 26th of February following, he experienced a chill, with peculiar symptoms about the throat, as if he had "taken cold." These symptoms continued to grow worse during the next twenty-four hours, when, at noon the following day, he expired from an attack of convulsions. Scarcely any favorable change, even temporarily, was produced by the use of atropine, ice to the spine, and enemata of oil and infusion of tobacco. Patient died under the influence of chloroform, at 11 o'clock P.M., February 28th.

### EXSECTION OF THE HEAD OF THE FEMUR.

Dr. Bogue exhibited the upper portion of the femur, four inches in length, which he had removed from a male patient at the County Hospital. The patient, 26 years of age, had enjoyed perfect health till ten months previous to the operation. On recently entering the hospital, there was a large swelling, with fluctuation on the anterior and upper portion of the thigh, encroaching somewhat upon the walls of the abdomen. The thigh was slightly flexed; the least motion produced excessive pain. The affection could not be traced to any violence, or to any apparent diathesis.

A free incision into the abscess was followed by the discharge of a pint and a-half of pus. No communication could be found between this abscess and the cavity of the joint. Exsection was performed in the usual manner. The upper portion of the femur was found extensively roughened and carious, as shown in the specimen. The ligamentum teres was destroyed, as also the upper and outer portion of the brim of the acetabulum.

### EXTRACTION OF A HARD CATARACT WITH A SOMEWHAT UNUSUAL COMPLICATION.

Dr. Holmes exhibited a hard cataract, which had been removed from a patient in the Chicago Charitable Eye and Ear Infirmary, 75 years of age, under the following, somewhat unusual, circumstances:—The cataract had been slowly forming for the past five years, during the last two of which the patient could only distinguish light and shade. At the first examination, a year ago, both eyes were found in a peculiar condition: the pupils were exceedingly minute and quite irregular, the whole pupillary edge of each iris being so firmly adherent to the opaque lens that the free use of strong solution of atropine failed to produce the slightest effect upon the pupils. There had never been the slightest pain, irritation or redness in the eyes, indicative of iritis. The irides were not pressed forward by aqueous humor behind them; they presented a peculiar pale blue color, as if their pigment had been absorbed. The patient could perceive the light from a candle in the dark, at the distance of twenty-five feet.

An extensive iridectomy downward on the left eye was performed, the patient being under the influence of chloroform. After the operation, it was found that the pupillary edge of the iris still remained attached to the opaque lens throughout its whole extent. There were, in fact, two pupils, the natural pupil and the artificial one below it, the two being separated by a narrow band of iris. For various personal reasons, the patient did not return to the city for the extraction till a year after the iridectomy.

The extraction was performed in the usual manner, the section of the cornea being made downward with the ordinary cataract knife. No chloroform was administered on account of the most alarming arrest of respiration during its use at the preliminary operation of iridectomy. Every portion of the cataract, the hard nucleus, the indurated cortical substance, together with the capsule, escaped in a mass, without the loss of the slightest quantity of vitreous humor. The cortical substance was much harder than was anticipated, which condition caused considerable difficulty in bringing the lens through the cornea. Yet the narrow band between the natural and artificial pupils was not ruptured.

For the first forty-eight hours there was somewhat more pain than usual, after extraction of cataract. Although considerable conjunctivitis supervened, the patient was able at the expiration of six weeks to walk comfortably alone in the streets, the eye being shaded, and to read coarse print (Snellin's test letters, No. 7) at the distance of a foot, without the aid of a lens. It is, perhaps, particularly worthy of remark, that during the seventh and eighth weeks after the operation, both the normal and artificial pupils were slowly contracting in size. The normal-pupil, at the same time, became elongated and drawn downward. There was no pain, and the conjunctivitis had nearly subsided. This contraction of the pupil may possibly be a result of the same process by which the iris became primarily attached to the lens. The patient was directed to return after a few months, for the purpose of providing himself with a suitable lens to correct the abnormal refraction and a marked astigmatism which exists in the cornea.

CASE OF OVARIOTOMY—RECOVERY.

Dr. Paoli read the very interesting history of the successful removal of an ovarian tumor, as given on another page of this journal.

